# Effects of High Temperature and Water Stress on Seed Germination of the Invasive Species Mexican Sunflower

**DOI:** 10.1371/journal.pone.0141567

**Published:** 2015-10-28

**Authors:** Bin Wen

**Affiliations:** Center for Integrative Conservation, Xishuangbanna Tropical Botanical Garden, Chinese Academy of Sciences, Menglun, Mengla, Yunnan, 666303, China; Universidad Nacional Autonoma de Mexico, MEXICO

## Abstract

Mexican sunflower is native to Mexico and Central America and was introduced into China early last century. Now it has widely naturalized and is exhibiting increasing invasiveness in South China. As this species often dominates bare ground, a habitat characterized by extreme fluctuation in temperature and water, it is reasonable to hypothesize that it has special adaptations to high temperature and water stress. Using laboratory experiments to simulate these stresses, this study investigated the response of Mexican sunflower seed germination to temperature and water stress, and compared these responses with those previously reported for another invasive, bamboo piper, which is confined to relatively cool and moist habitats in Xishuangbanna. As expected, Mexican sunflower seeds exhibited higher tolerance to these stresses than bamboo piper. Germination of Mexican sunflower seeds was highest at 15–30°C, but significant numbers of seeds germinated and formed seedlings at 10°C and 35°C, at which no bamboo piper seeds formed seedlings, indicating a wider temperature range for germination than the latter. Roughly half the seeds survived 240 h continuous heat treatment and up to 15 h daily periodical heat treatment at 40°C, while bamboo piper seeds were mostly killed by these treatments. About 20% of Mexican sunflower but no bamboo piper seeds germinated after heat treatment for 30 min at 80°C. Germination was completely inhibited in bamboo piper seeds at -0.6 mPa, while 20–60% of Mexican sunflower seeds germinated depending on PEG or NaCl as osmoticum. This higher tolerance in Mexican sunflower seeds accords with its stronger invasiveness in this area. This comparison between two plant invaders demonstrates that invasiveness is not an all-or-nothing situation, and that adaptation to local habitats is a critical determinant of successful invasiveness for an alien plant.

## Introduction

Mexican sunflower (*Tithonia diversifolia* (Hemsl.) A. Gray) in the family Asteraceae is a weed native to Mexico and Central America. It has been introduced widely into Asia, Africa, America and Australia, as an ornamental plant for its beautiful tall flowers, and as a green manure and erosion-control plant for its fast growth. Now it has become an aggressive invader in Southeast Asia, South Africa and many Pacific islands [1–4]. This species was found escaped in Yunnan, China, in the 1930s, but has behaved as an aggressive invader only from around 2000 [5, 6]. Now it is abundant in tropical China, including Fujian, Guangdong, Hainan, Guangxi, Yunnan and Taiwan [4].

Mexican sunflower is described as an herbaceous stoloniferous annual in its native range, but in Xishuangbanna it grows as a shrubby perennial with numerous woody upright branching stems up to 5 m high. Here it invades roadsides, disturbed areas and bare ground, forms dense stands, preventing the growth of young native plants, and causes damage to agricultural production and biodiversity conservation [4, 7]. Both Mexican sunflower and the previously-studied bamboo piper (*Piper aduncum* L.) [8] are alien plants of Central American origin in Xishuangbanna, but Mexican sunflower differs from bamboo piper, which dominates only in relatively damp, semi-shaded habitats, such as forest edges, in its ability to colonize and dominate in dry, exposed sites with full sun.

As seed germination is a critical developmental phase change in plant life cycle and playing important roles in seedlings establishment and environmental adaptation [9], its adaptation to habitats may be a critical determinant of successful invasiveness for an alien plant. In this aspect, bamboo piper and Mexican sunflower form a good comparison, since the habitats occupied by Mexican sunflower have more extreme fluctuations in temperature and water availability than those occupied by bamboo piper. If seed germination plays an important role in their invasiveness [10,11,12], then we expect that Mexican sunflower seeds, which can establish on bare ground, will have broader tolerance to high temperatures and water stress than bamboo piper seeds, which do not establish under these conditions. In this study, laboratory experiments were conducted to investigate the effects of high temperature and water stress on seed germination in the invasive species Mexican sunflower, and the results are compared those previously obtained from bamboo piper.

## Materials and Methods

### Seed Collection and Pretreatment

In Xishuangbanna, Mexican sunflower begins flowering in October every year, achieves its peak bloom in November to December, and mature achenes (hereafter called seeds, although technically they are dry fruits) are shaken off by wind in January to March the following year. These seeds pass through March and April, the hot dry season, on the ground, before their germination requirements are met during the rainy season, from May to October [5, 7].

Seeds used in this study were collected from Menglun, where Xishuangbanna Tropical Botanical Garden, Chinese Academy of Sciences (21°55' N, 101°15'E) is located, in February 2015. After collection, seeds were air-dried in ambient conditions for a few days, with debris removed, and then initial moisture content and seed weight were determined, and initial viability assessed using methods described below. The remaining seeds were sealed in a polyethylene bag and kept at 15°C.

The seed had been stored at 15°C for about 2–3 months before the following experiments were performed in April-June 2015. After withdrawal from storage conditions, the seeds were first dried with silica gel for two days and then rehydrated over saturated air at 30°C for 24 h. A preliminary test found that this pretreatment can greatly improve seed germination, from 30% to 90%.

### Experimental Arrangements

In order to provide a comparison with bamboo piper, the experimental design in this study largely followed the previous study [8], with only very limited modifications.

#### Effects of relative humidity on seed moisture and viability

An eRH spectrum between 8 and 85% was created using eleven saturated salt solutions (KOH, K acetate, MgCl_2_, K_2_CO_3_, Mg(NO_3_)_2_, NaNO_2_, CuCl_2_, NaCl, NH_4_Cl, (NH_4_)_2_SO_4_, KCl) in sandwich boxes. Seeds were firstly equilibrated over these solutions for 2 weeks at 25°C, and then taken out for moisture content and viability determination. The low-temperature oven method recommended by International Seed Testing Association [13] was used, i.e., oven-drying samples of 10–15 seeds at 103±2°C for 17 h, seed moisture contents were determined gravimetrically and expressed on the wet weight basis as means ± SE of 8 replicates.

#### High-temperature tolerance of quiescent seeds

To determine the effect on viability of extreme high temperature, such as seeds might experience during a hot summer’s day after being dispersed to bare ground, a temperature range from 30°C to 95°C provided by a water bath was employed as in the previous study [8]. For each testing temperature, two triangular flasks containing around 350 seeds each were used. The seeds in one flask were kept air-dried during heating while those in the other were moistened half an hour before heat treatment. After heating for half an hour at the indicated temperature, the seeds were sown for viability assessment.

#### Effects of incubation temperature and light on seed germination

Seeds sown on 1% (w/v) water agar in Petri dishes were placed in incubators set at constant temperatures of 10 to 40°C at 5°C increments, and alternating temperature of 18/28°C, with a 12 h photoperiod of 25 μ mol m^-2^ sec^-1^ irradiance provided by a cool white fluorescent light.

For the dark treatment, Petri dishes with seeds were wrapped in two layers of aluminum foil to ensure no light penetration. Germination in the dark was viewed by ground illumination from a green safe light once a week.

#### Effects of water availability on seed germination

Solutions with water potentials of -0.05, -0.10 to -1.2 mPa were created by polyethylene glycol (PEG) 8000 according to Michel [14] and NaCl according to Lang [15], respectively, and a control treatment (pure de-ionized water, 0 mPa) was included. Filter paper discs (medium speed, qualitative) saturated with 1.5 ml of de-ionized water or treatment solutions were used as germination medium. Seeds sown on moistened filter paper were incubated at 25°C in light for germination. The six Petri dishes with the same treatment solutions were sealed in a resealable double-clear plastic bag to minimize moisture loss during the experiment. Every other day, these Petri dishes were taken out to change the germination medium, and seed germination checked. Six weeks later, ungerminated seeds were removed from stress, washed using de-ionized water and placed at 25°C in light for viability assessment.

In addition, the effects of soil moisture on seed germination were investigated. A laterite soil, the typical soil with a deep solum and thin humus horizon which occurs in Xishuangbanna at altitudes between 600 and 1000 m, was sampled from bare ground. The soil sample was first air-dried for a few days, and then crushed into small grains (≤ 0.2 mm), with the litter removed, before drying at 70°C to constant weight. Fifty grams of this dried soil with 5–20 g de-ionized water was dispensed into 250 ml glass jars to create soil moistures in the range 10–40% (dry weight basis). After sowing 50 seeds in each, these jars were sealed with a plastic membrane and kept in ambient conditions with natural light for germination.

#### Effects of continuous heat treatment on seed viability

To investigate the tolerance of Mexican sunflower seeds to a continuous heat treatment, seeds were sown on water agar as previously described and placed in an incubator at 40°C. After heat treatment for a given period of time, they were withdrawn from the incubator and placed under ambient conditions (air-conditioned at 25°C and normal light) to assess seed viability.

#### Effects of daily periodic high temperature on seed germination

In this experiment, seeds were sown on water agar as previously described and were physically transferred into and out of a 40°C incubator daily so that they were exposed to alternations of 1h/23h, 2h/22h, 3h/21h, 5h/19h, 7h/17h, 9h/15h, 12h/12h and 15h/9h between 40°C and ambient temperature (air-conditioned at 25°C) to investigate seed germination under periodic heat stress.

#### Desiccation interruption during seed germination

In order to investigate effects of imbibition-dehydration treatment, such as what might happen to seeds during a sunny day after a storm, seeds were sown on filter paper moistened with de-ionized water in Petri dishes and placed into an incubator at 25°C with light conditions as described above. After incubation for a given period of time (up to 120 h), they were sampled, and those with visible germination were scored and kept to germinate. The ungerminated seeds were removed and air-dried at 50% RH and 15°C for 72 hours. After these interruptions, they were re-imbibed on moistened filter papers and put back into a 25°C incubator for viability assessment.

### Seed Viability and Germination Assessment

Fifty seeds × 6 replicates were used for each treatment in this study. Unless stated otherwise, the seeds were scored once a week for at least 6 weeks, those with visible germination (i.e. radicle emergence) were considered to have germinated, or survived for stress-treated seeds, and formation of normal seedling from these seeds was also assessed. A crush test was employed on any non-germinated seeds to confirm that they were non viable before finishing the experiments.

### Data Analysis

Data are presented as means and standard errors, and subjected to one- or two-way ANOVA and Duncan’s multiple comparison tests (α = 0.05) after arc-sine transformation, using SPSS 13.0 for Windows.

## Results

Mexican sunflower seeds used in this study had a 100-seed weight of 442.48±3.29 mg, an initial moisture content of 9.00±0.38% (wet weight basis), and an initial germination of 92.33±1.84%. Almost all germinated seeds developed into normal seedlings in the experiments, so only germination percentage is reported in this study.

### Effects of relative humidity on seed moisture and viability

**Fig 1:**
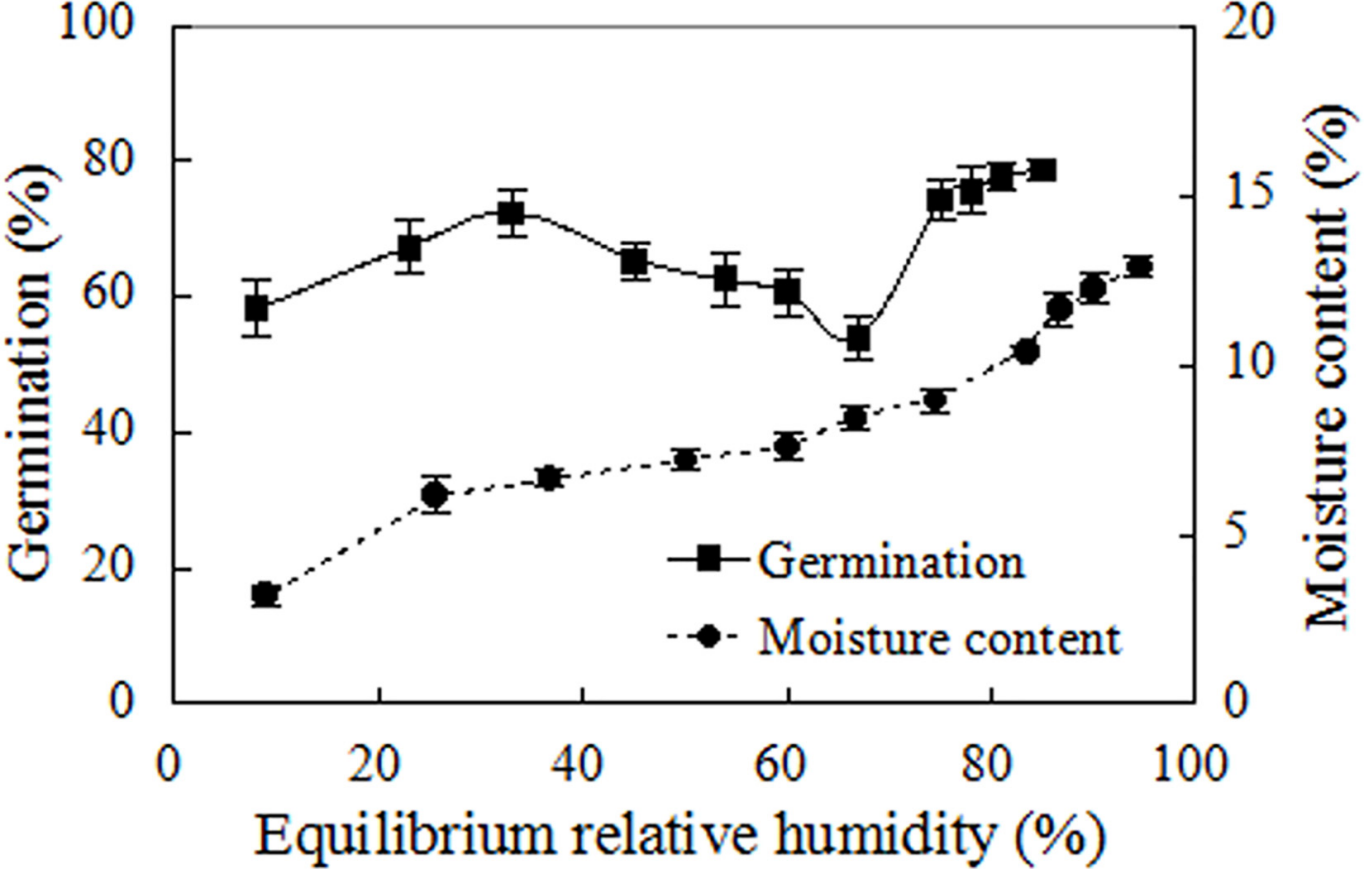
Effects of equilibrium relative humidities from 8% to 85% at 25°C on seed germination and moisture contents. Germination values are means±SE of 6 replicates of 50 seeds.

Equilibration treatment over the eleven saturated salt solutions changed not only seed moisture contents, but also their germination (Fig 1). After two week’s equilibration, seed moisture contents ranged from 3% to 13%, with higher seed moisture contents corresponding to higher relative humidities. Interestingly, seed germination demonstrated a V-shaped response to equilibration treatment under relative humidity (F = 6.673, *p*<0.001), with the minimum germination occurring at 67% equilibrium relative humidity. To understand the mechanism for this response requires further study, but it may be caused by seed deterioration or dormancy induction, or both.

### High-temperature tolerance of quiescent seeds

**Fig 2:**
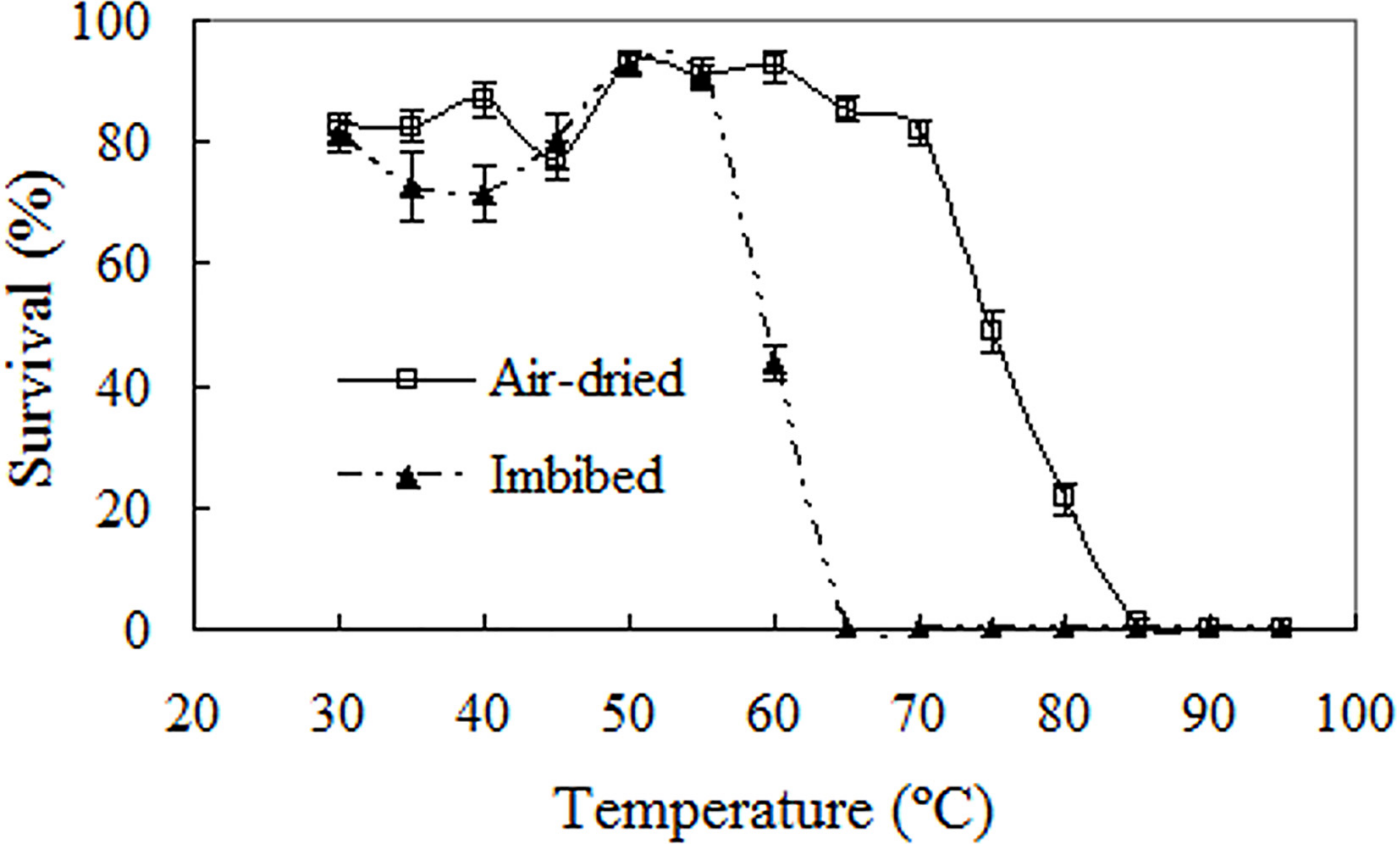
Effects of half an hour heat shocks from 30°C to 95°C on viability of air-dried or imbibed seeds. Germination values are means±SE of 6 replicates of 50 seeds.

As predicted, Mexican sunflower seeds exhibited high tolerance to extreme high temperature, and heat treatment at 50–55°C actually raised the germination percentage (Fig 2). Air-dried seeds had no marked viability loss after shock at temperatures up to 70°C, and half the seeds survived 75°C. Although 85°C killed all seeds, one fifth of seeds germinated and formed morphologically normal seedlings after heat treatment for 30 min at 80°C (Fig 2). Both temperature and seed hydration status had significant effects on seed viability (two-way ANOVA; *p*<0.001 for heat temperature, seed hydration, and heat temperature×seed hydration). Imbibed seeds were considerably more sensitive to heat: only 40% germinated after heating for 30 min at 60°C and no seeds survived 65°C and higher temperatures.

### Effects of incubation temperatures and light on seed germination

**Fig 3:**
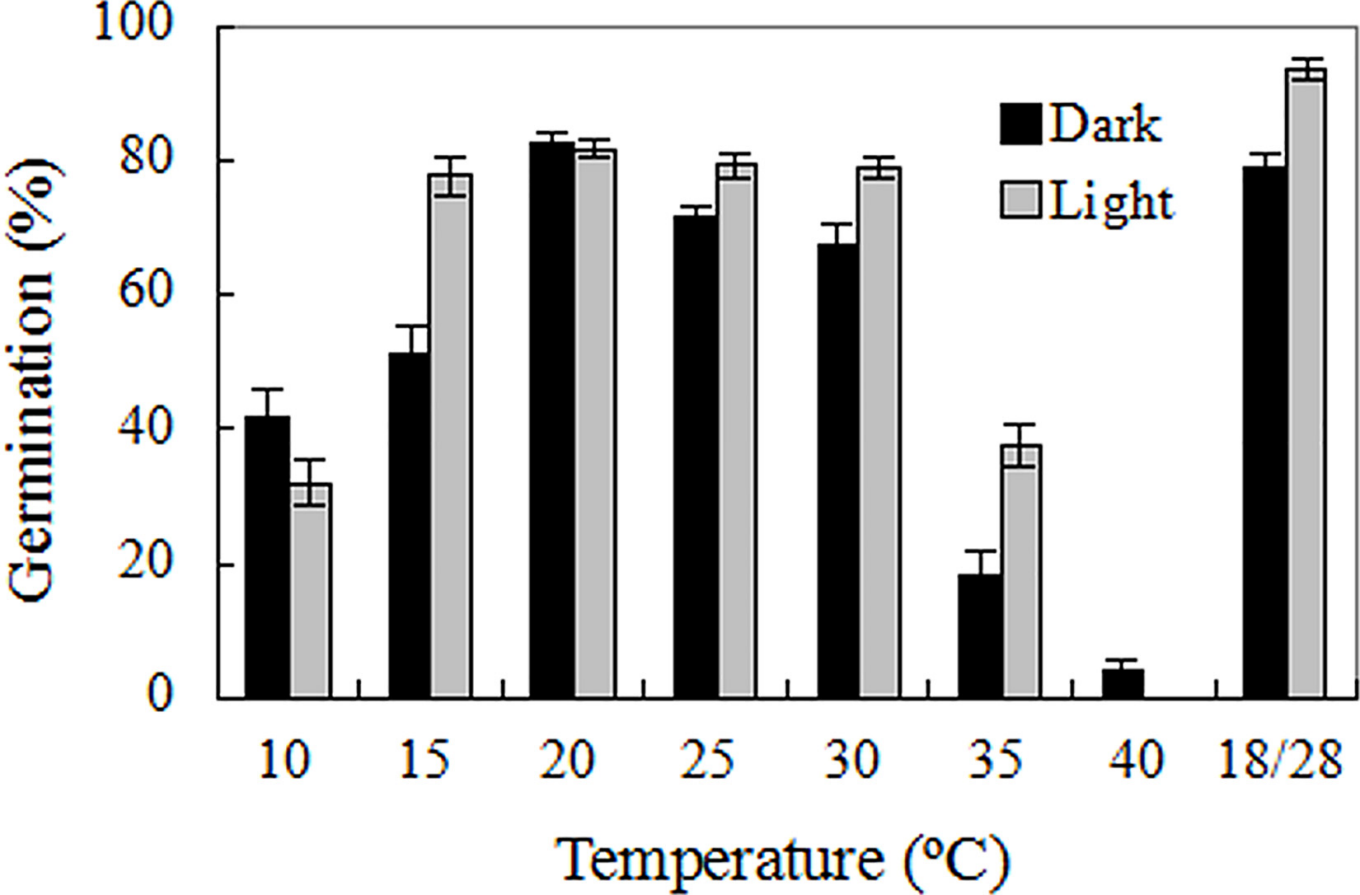
Effects of incubation temperatures and light on seed germination. Seeds were incubated at constant temperature from 10°C to 40°C, and 18/28°C. Germination values are means±SE of 6 replicates of 50 seeds.

Mexican sunflower seeds are non-photoblastic, reaching germination percentages higher than 80% in both light and dark conditions, but both temperature and light conditions influenced germination (two-way ANOVA; *p*<0.001 for incubation temperature, light condition, and incubation temperature×light condition). Seed germination preferred a temperature regime of 15–30°C, and fluctuating temperature 18/28°C gave the maximum germination, but significant numbers (20–40%) of seeds germinated at 10°C and 35°C. Dark germination was higher than light germination at 10°C and 40°C while this did not happen in the other temperature regimes tested (Fig 3).

### Effects of water availability on seed germination

**Fig 4:**
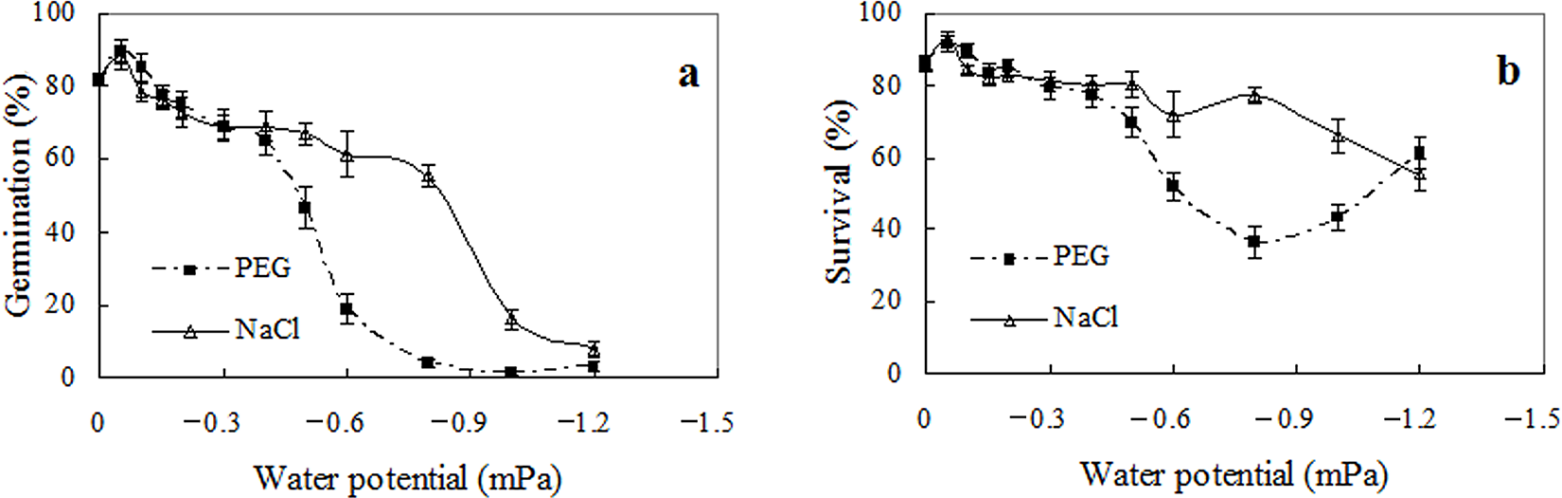
Effects of water potentials on seed germination. Seed germination were calculated after incubation for 6 weeks under water stress (a), and 4 more weeks after release from stress (b). Germination values are means±SE of 6 replicates of 50 seeds.

Both the water potential of the germination medium and the reagents used to reduce water potential had significant effects on germination (two-way ANOVA; *p*<0.001 for water potential, reagent, and water potential×reagent). A water potential of -0.3 mPa significantly reduced germination compared with the control (de-ionized water, 0 mPa). Under stress of equal water potentials between -0.5 and -1.0 mPa, seeds treated with PEG solutions had lower germination than those with NaCl solutions. Germination was depressed to 20% at -0.6 mPa created by PEG, or -1.0 mPa by NaCl (Fig 4A).

However, many ungerminated seeds germinated when released from water stress after 6 weeks incubation in the test solution, showing that they were viable, but germination was inhibited, which was very obvious when water stress is larger than -0.6 mPa (Fig 4B).

**Fig 5:**
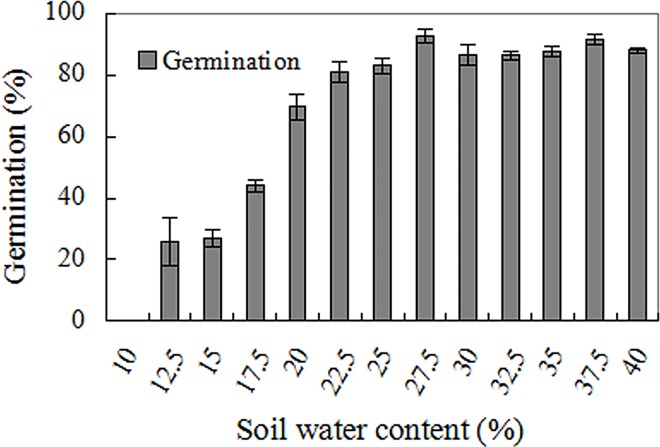
Effects of soil moisture on seed germination. Each glass jar contained 50 seeds and 50 g local soil with varying soil moisture. Germination values are means±SE of 6 replicates of 50 seeds.

Mexican sunflower seeds germinated within a wide soil water range, although low soil water content inhibited germination (F = 4.735, *p*<0.001). About a quarter of seeds germinated at soil water contents as low as 12.5%. Germination percentage rose to 40% and 80% when soil water content reached 17.5% and 22.5%, respectively (Fig 5).

### Effects of continuous heat treatment on seed viability

**Fig 6:**
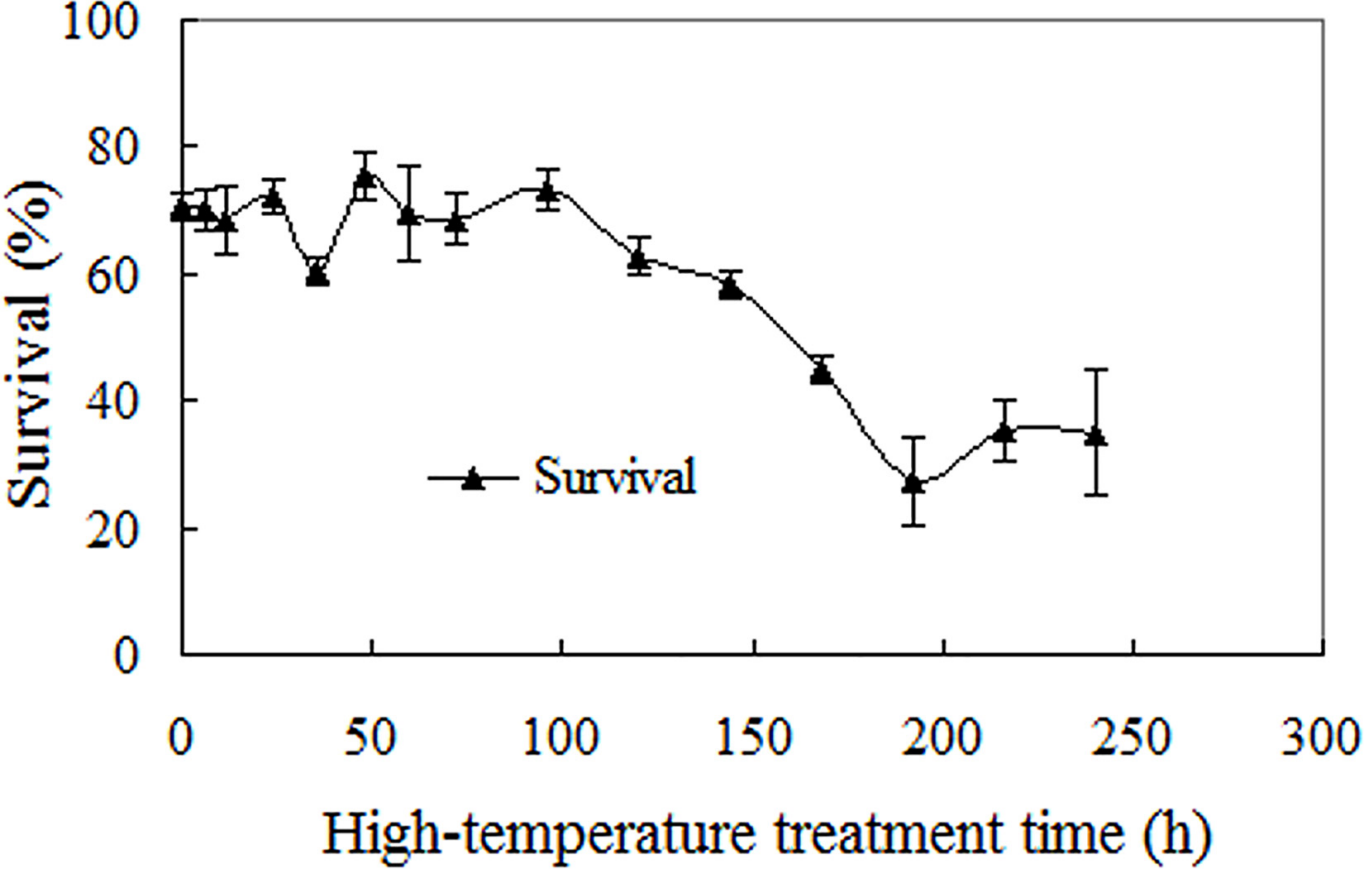
Effects of continuous high temperature stress on seed germination. Seeds were heat-shocked for a given period of time at 40°C, and incubated at ambient conditions after released from stress. Germination values are means±SE of 6 replicates of 50 seeds.

Duration of continuous heat treatments at 40°C influenced germination of Mexican sunflower seeds (F = 10.032, *p*<0.001), but had no significant effect on germination percentage until it reached 168 h. Around 30–40% of Mexican sunflower seeds still survived even after a 240 h heat treatment at this temperature (Fig 6).

### Effects of periodic high temperature on seed germination

**Fig 7:**
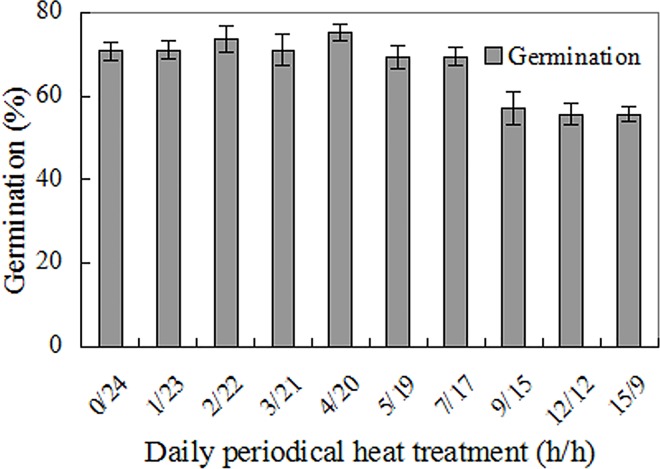
Effects of daily periodic high temperature stress on seed germination. 40°C high temperature and ambient temperature (h/h) were imposed on seeds alternately. Germination values are means±SE of 6 replicates of 50 seeds.

Compared to the control (0 h heating), up to 7 h daily heat treatments at 40°C had little impact on germination. But 9–15 h daily heat treatments made a significant reduction (F = 7.059, *p*<0.001), however, 60% of seeds still germinated (Fig 7).

### Effects of desiccation interruption on seed germination

**Fig 8:**
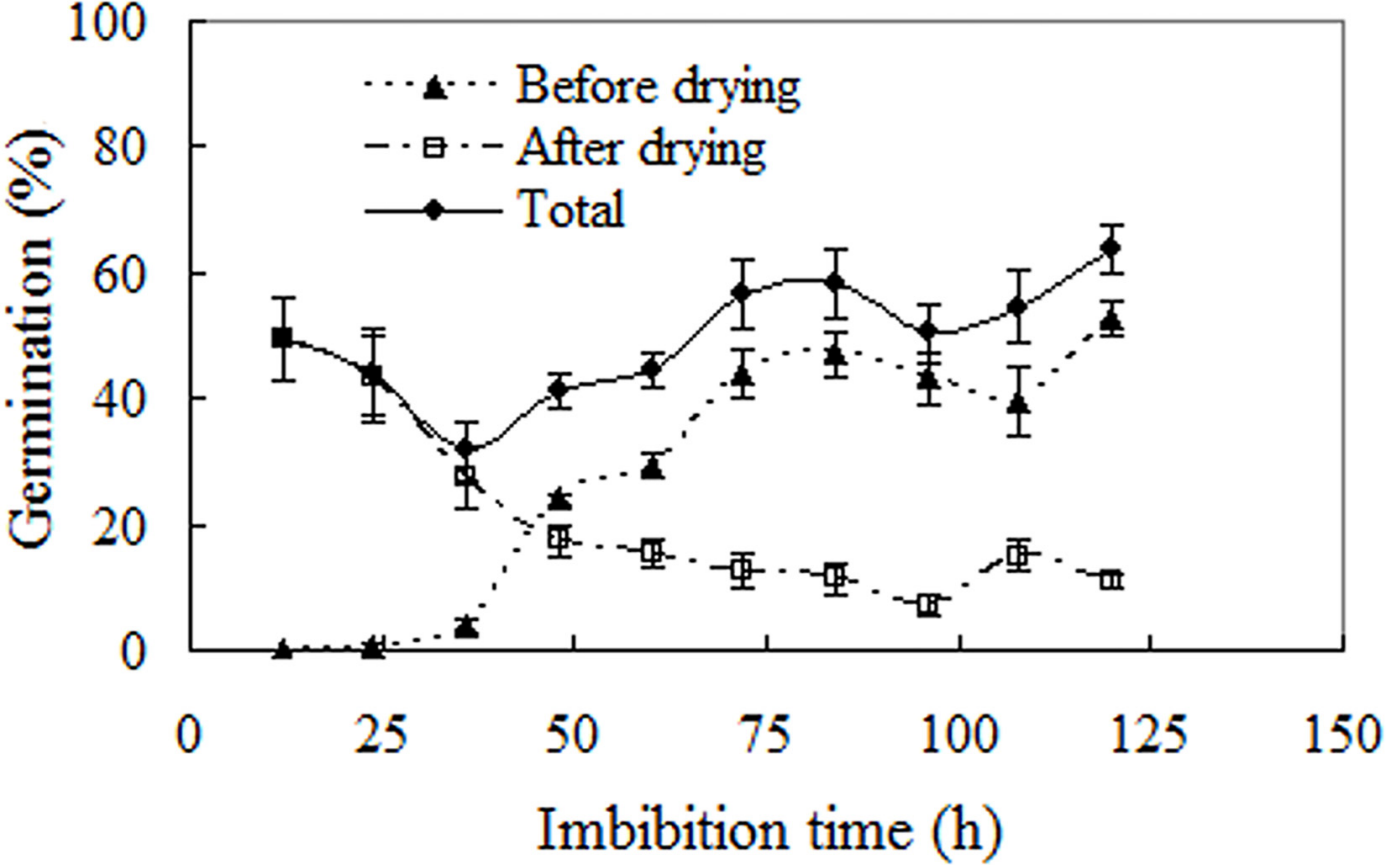
Effects of dehydration after imbibition on seed germination. After imbibition for the indicated time, the seeds were dried at 15°C under 50% eRH for 72 h, and then re-imbibed. Germination values are means±SE of 6 replicates of 50 seeds.

Compared with the control (80±2.58% germination percentage for seeds without desiccation interruption, data not shown), desiccation interruption during germination markedly reduced total germination percentage, with the reduction in amplitude depending on imbibition time prior to desiccation (F = 3.455, *p* = 0.002; Fig 8). For Mexican sunflower seeds 36-hour’s imbibition is a critical stage. Firstly, it was the earliest stage for visible germination to be found; secondly, putting pre- and post-desiccation germination together, seeds desiccated following 36-hour’s imbibitions gave the lowest total percentage germination (about 25%); finally, 36-hour’s imbibition followed by desiccation is a turning point, before this point all germination happened after desiccation, after this point most germination happened before desiccation. Whenever desiccation is interposed, at least 25% germination percentage can be reached (Fig 8).

## Discussion

Following a previous study on bamboo piper seeds [8], this paper investigated germination of Mexican sunflower seeds under high temperature and water stress, and found that Mexican sunflower seeds have markedly higher abiotic tolerance than bamboo piper seeds. Although water stress conditions restricted seed germination in both species, a germination percentage of 20–60% was achieved by Mexican sunflower at -0.6 mPa (Fig 4) while no germination occurred in bamboo piper at this water potential [8]. In addition, about 25% of Mexican sunflower seeds germinated in soil as dry as 12.5% moisture content (dry weight basis, Fig 5). Similarly for high temperature stress, a significant number of seeds germinated although high temperature also reduced seed viability and impaired seed germination in Mexican sunflower. After heat treatment for 30 min at 80°C, all bamboo piper seeds were killed [8] while 20% of air-dried Mexican sunflower seeds survived and developed into normal seedlings (Fig 2). Moreover, Mexican sunflower seeds can germinate at higher incubating temperatures. When incubated at 35°C, 20–40% of Mexican sunflower germinated and formed normal seedlings (Fig 3) but no seedlings formed for bamboo piper [8]. Few bamboo piper seeds formed seedlings when the duration of continuous heat treatment at 40°C increased to 240 h, or the period of time for daily periodical heat treatment reached 12 h [8], while these treatments only partially impaired seed germination and seedling establishment in Mexican sunflower (Figs 6 and 7). Compared with bamboo piper, Mexican sunflower seeds germinated across a wider range of environmental conditions.

Biological invasion is an increasingly serious problem, and has attracted considerable attention worldwide [10]. Whatever the cause, biotic invaders can in many cases inflict enormous environmental and agriculture damage [16]. Through competition, habitat alteration and out-competing native species, plant invaders can greatly diminish the abundance or survival of native species and threaten biodiversity in native ecosystems, meanwhile causing crop losses and increased pest control costs. Thus understanding the mechanisms of plant invasions is crucial to weed species management and predicting future invasion patterns [17, 18]. For this purpose, two complementary approaches were frequently used in previous studies: one is to study the invading species in an attempt to identify the characteristics that enable it to invade new areas, particularly the response to perturbation in cultivated areas; the other is to describe the habitat that a species has invaded in order to try to establish the factors making that habitat susceptible to invasion [19]. These studies usually use yes or no to discriminate invasiveness of a plant attribute and invasibility of a habitat.

Both Mexican sunflower and bamboo piper are native to Mexico and Central America, and have become plant invaders in Xishuangbanna. As invasive species, they are both prolific seed producers, with the capability to produce small but numerous viable seeds every year, have rapid growth, high rates of biomass accumulation, a short juvenile period and high reproductive efforts [2, 7, 20–22], all attributes which are thought to contribute to invasiveness [19, 23, 24]. On the other hand, deforestation and habitat disturbance in this area in past decades [25] increased resource availability for these invaders, for these changed biotic and abiotic conditions [26–30], and are widely recognized as a primary influence to make the habitat more susceptible to invasion, or even become a crucial driver of invasion [31–35]. Although its seeds are small and strict positively photoblastic [20], bamboo piper dominates only relatively cool and wet habitats like forest ridges and remains an intermediate invader in Xishuangbanna [8] while Mexican sunflower can colonize bare ground and form monospecific stands, and demonstrates stronger invasiveness in this area [4,7].

The results in this study have several implications. Firstly, they suggest that higher tolerance in seeds to high temperature and water stress may have helped confer the species’ stronger invasibility. Indeed, this study found higher stress tolerance in Mexican sunflower seeds, which germinated better under high temperature and water stress than previous-reported bamboo piper seeds [8], so it can be expected that Mexican sunflower seeds, with the capability of germination under many conditions, are more likely to spread into and colonize new habitats than bamboo piper seeds, with relatively strict germination requirements. Secondly, invasion success is the consequence of interaction between alien plants and invaded areas, reflecting the fitness of the species to the habitats which is not an all-or-nothing situation. In Xishuangbanna, for example, chromolaena (*Chromolaena odorata* (L.) King & Robinson) [36] and Mexican sunflower are aggressive invaders while bamboo piper is intermediate [8]. Of course, this does not mean that bamboo piper must score lower than Mexican sunflower in every aspect related to invasiveness as discussed later, nor can it preclude the possibility for bamboo piper to be an aggressive invader under certain conditions: it is reported to grow in open sites and form monospecific stands in Malaysia and Kalimantan, where there is no dry season [37]. Finally, Mexican sunflower and bamboo piper are both potential invaders, but they occupy different microhabitats in Xishuangbanna, indicating the importance of microhabitat conditions for successful invasion. These are in accord with the existing theories on plant invasiveness. For example, Pyšek and Richardson [11] proposed that germination of alien invasive species was more rapid, higher and successful across more environmental conditions than that of congeneric native⁄noninvasive taxa, and Wainwright and Cleland [12] suggested that exotic species have more rapid and prolific germination across a variety of environmental cues and in response to increased resource availability compared with native species, i.e., exhibit both germination plasticity and robustness.

Invasibility is a complex trait, for colonization success is determined by many factors, with germination success in a range of environments being one of several traits to be associated with plant invasiveness [38]. The coincidence of stronger invasibility with higher seed germination under high temperature and water stress in Mexican sunflower provided supports for the physical environmental mechanism of biological invasion [18], however, this does not exclude contributions of other factors and mechanisms, for example, allelopathic effects on plants, the so-called ‘novel weapon’ commonly associated with plant invaders [39], which has been reported in Mexican sunflower [3, 40], but not in bamboo piper as yet, also could facilitate successful colonization of Mexican sunflower in new areas. On the other hand, Mexican sunflower also possesses traits making it score low in invasiveness relative to bamboo piper, for example, the seeds are much larger than those of bamboo piper, with 100-seed weight of 442.48±3.29 mg v.s. 18.05±0.86 mg [8], and are shed in the dry and hot season [7], while bamboo piper has indeterminate flowering and mature seeds are released most of the year [8, 21, 41]. Hence, invasiveness of Mexican sunflower is determined by multiple factors, including those that both favor and disfavor invasiveness, and these need comprehensive assessment or incorrect conclusions may be drawn.
